# Supplementary material for: In Vitro Immunological Cross-Reactivity of Thai Polyvalent and Monovalent Antivenoms with Asian Viper Venoms
Source: Toxins (Basel). 2020 Dec 3;12(12):766. doi: 10.3390/toxins12120766 (PMC7761867; doi:10.3390/toxins12120766)
Supplement: Supplementary file 1 [file toxins-12-00766-s001.pdf]

Supplementary Materials: In Vitro Immunological Cross-Reactivity of Thai Polyvalent and Monovalent Antivenoms with Asian Viper Venoms

Janeyuth Chaisakul, Muhamad Rusdi Ahmad Rusmili, Jaffer Alsolaiss, Laura-Oana Albulescu, Robert A. Harrison, Iekhsan Othman and Nicholas R. Casewell

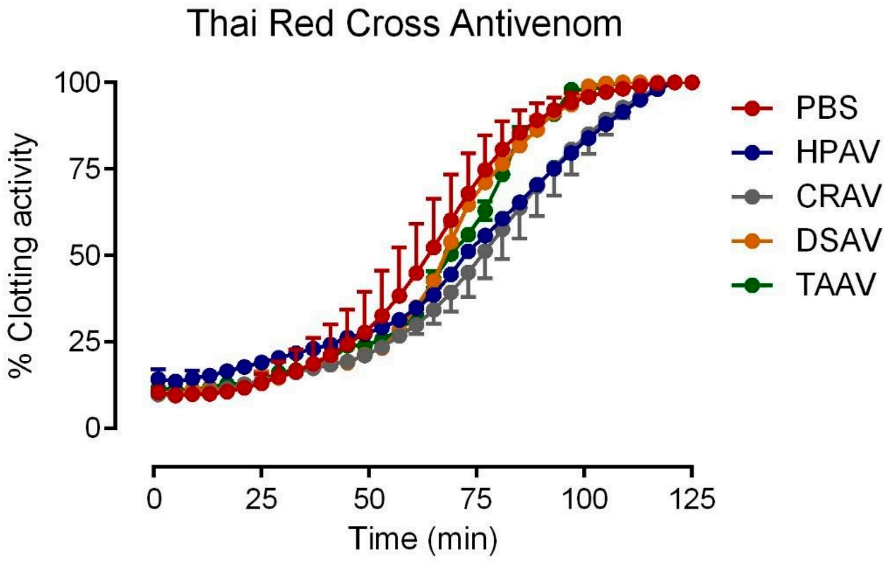

Figure S1. Clotting activity (%) vs Time (min) of bovine plasma in the absence (PBS; i.e. normal coagulation) and presence of the Thai Red Cross antivenoms used in this study (i.e. HPAV; Hemato Polyvalent antivenom, CRAV; *Calloselasma rhodostoma* antivenom, DSAV; *Daboia siamensis* antivenom and TAAV; *Trimeresurus albolabris* antivenom).
